# Association of blood manganese level with diabetes and renal dysfunction: a cross-sectional study of the Korean general population

**DOI:** 10.1186/1472-6823-14-24

**Published:** 2014-03-08

**Authors:** Eun Sil Koh, Sung Jun Kim, Hye Eun Yoon, Jong Hee Chung, Sungjin Chung, Cheol Whee Park, Yoon Sik Chang, Seok Joon Shin

**Affiliations:** 1 Department of Internal Medicine, College of Medicine, The Catholic University of Korea, 222 Banpo-daero, Seoul 137-701, Republic of Korea; 2 Division of Nephrology, The Catholic University of Korea Yeouido St. Mary’s Hospital, 10, 63-ro, Yeongdeungpo-gu, Seoul 150-713, Republic of Korea; 3 Division of Nephrology, The Catholic University of Korea Incheon St. Mary’s Hospital, 56, Dongsu-ro, Bupyeong-gu, Incheon 403-720, Republic of Korea; 4 Department of Statistics, The Graduate School of Ewha Womans University, 52, Ewhayeodae-gil, Seodaemun-gu, Seoul 120-750, Republic of Korea; 5 Division of Nephrology, The Catholic University of Korea Seoul St. Mary’s Hospital, 222, Banpo-daero, Seoul 137-701, Republic of Korea

**Keywords:** Manganese, Diabetes, Renal dysfunction

## Abstract

**Background:**

The purpose of this study was to evaluate the association between blood manganese levels and the prevalence of chronic diseases in the Korean population.

**Methods:**

This was a cross-sectional study based on the Korean National Health and Nutrition Examination Survey (KNAHNES). The study included 3996 participants 20 years of age or older whose blood manganese levels had been measured. The participants were also evaluated for the presence of five chronic diseases: diabetes, renal dysfunction, hypertension, ischemic heart disease, and stroke.

**Results:**

Blood manganese levels were significantly lower in the diabetes group compared with the non-diabetes group (1.26 ± 0.02 vs. 1.35 ± 0.01 μg/dL; *p* = 0.001) and the renal dysfunction group compared with those with normal renal function (1.28 ± 0.03 vs. 1.35 ± 0.01 μg/dL; *p* = 0.04). There was no significant association between blood manganese levels and the presence of ischemic heart disease or stroke. A multivariate logistic regression analysis adjusted for age, sex, and body mass index was performed; the odds ratio was 0.652 (95% CI: 0.46–0.92) for diabetes and 0.589 (95% CI: 0.39–0.88) for renal dysfunction when comparing the higher quartiles (Q2-4) with the lowest quartile (Q1) of blood manganese level. The prevalence of diabetes was 7.6% in Q1 and 5.3% in Q2-4 (*p* = 0.02). Similarly, the prevalence of renal dysfunction was 6.8% in Q1, compared with 4.6% in Q2-4 (*p* = 0.02).

**Conclusion:**

The prevalence of diabetes and renal dysfunction increased in participants with low blood manganese levels, suggesting that blood manganese may play a role in glucose homeostasis and renal function.

## Background

Manganese (Mn) is an essential trace metal with insufficient intake in virtually all diets. It has been reported that Mn is involved in normal immune functions, regulation of blood sugar and cellular energy, and the defense mechanisms against free radicals [1,2]. Experimentally induced Mn deficiency caused a number of detrimental effects, such as impaired bone formation, abnormal glucose tolerance, low levels of high-density lipoprotein (HDL) cholesterol, and skin abnormalities in both animals and humans [3]. Conversely, excess occupational inhalation of Mn may be neurotoxic to humans, producing effects such as psychosis and Parkinsonism. Mn deficiency and intoxication are both associated with adverse metabolic and neuropsychiatric effects [4,5]. Nevertheless, little is known about the optimal blood Mn levels for maintaining homeostasis in humans.

One study found that Mn inhalation in adult animal models markedly down-regulated the gene expression of proteins critical to inflammatory responses or possessing pro-oxidant properties (e.g. tumor growth factor (TGF)-β and neuronal nitric oxide synthase (nNOS)) [6]. These results suggested that a certain amount of Mn exposure may attenuate the inflammatory response to stressful environments. In contrast, Mn is an essential cofactor for metalloenzyme superoxide dismutase, which protects cells against antioxidant processes [3]. Malecki *et al.* demonstrated that Mn deficiency and the resultant decrease in manganese superoxide dismutase (MnSOD) activity caused heart mitochondria to be more sensitive to *in vivo* oxidative damage [7]. This study proposed that subclinical Mn deficiency might also contribute to excess oxidative stress.

Oxidative stress and inflammation are the main pathophysiology behind most chronic diseases. Therefore, we hypothesized that blood Mn levels could be related to the prevalence of chronic diseases. We conducted a cross-sectional analysis on the associations of blood Mn level with five chronic diseases (diabetes, renal dysfunction, hypertension, ischemic heart disease, and stroke) in a representative sample of the adult Korean population from the Korean National Health and Nutritional Examination Survey (KNHANES).

## Methods

### Study population

The KNHANES was performed to examine the general health and nutritional status of the civilian, non-institutionalized Korean population. KNHANES is a cross-sectional and nationally representative survey composed of a health questionnaire, a health examination, and a nutrition survey. The fourth KNHANES (KNHANES IV) was conducted from 2007 to 2009 using a stratified, multistage probability sampling design. We used data from the second (2008) to third year (2009) of the KNHANES IV. Of the 8641 individuals who participated during this time, we analyzed those who were 20 years of age or older and tested for blood Mn. We excluded participants who were pregnant or had missing values and yielded a final sample size of 3996 adults. The Catholic University of Korea Incheon St. Mary’s Hospital Institutional Review Board and the Korea Centers for Disease Control and Prevention approved the study protocol, and written informed consent was obtained from all participants before the study began.

### Laboratory measurements

Blood Mn was measured using whole blood at the Neodin Medical Institute (certified by the Korean Ministry of Health and Welfare) in Seoul, Korea, following a standardized protocol. Blood Mn was analyzed by graphite furnace atomic absorption spectrometry with Zeeman background correction (Perkin Elmer AAS800, Perkin Elmer, Turku, Finland). The limit of detection was 0.016 μg/dL for blood Mn. For internal quality assurance and control, standard reference materials were obtained from Bio-Rad (Lyphochek^®^ Whole Blood Metals Control). The inter-assay coefficients of variation ranged from 0.95% to 4.82% for blood Mn samples (reference values were 0.98, 1.18, 2.46, and 3.28 μg/dL). During the survey, overnight fasting venous blood samples were collected. The collected blood samples were properly processed, refrigerated, and transported in cold storage to the Neodin Medical Institute in Seoul, Korea.

### Chronic diseases

Diabetes was defined as having one of the following: a fasting blood glucose ≥ 126 mg/dL, a self-reported physician’s diagnosis, medication use, or insulin administration at the time of interview. Serum creatinine level was measured using a Hitachi Automatic Analyzer 7600 (Hitachi, Japan) and a modified kinetic Jaffe reaction. The level of kidney function was attained using an abbreviated equation developed from the data from the Chronic Kidney Disease-Epidemiologic Collaboration Group (CKD-EPI) study to estimate the glomerular filtration rate (GFR) [8]. We defined renal dysfunction as an estimated GFR (eGFR) of < 65 mL/min/1.73 m^2^[9]. Hypertension was defined as having one of the following: a mean systolic blood pressure of ≥ 140 mmHg, a mean diastolic blood pressure of ≥ 90 mmHg, a self-reported physician’s diagnosis, or antihypertensive medication use at the time of interview. Ischemic heart disease and stroke were based on a self-reported physician’s diagnosis.

### Other variables

Information on age, sex, residential area, educational status, smoking exposure and alcohol consumption, occupation, body mass index (BMI), and nutritional intake were based on a health questionnaire. Residential area was categorized as either urban or rural. Seoul (the capital city of South Korea) and six other metropolitan cities were grouped as urban areas, and the remaining regions were defined as rural areas. Educational status was divided into ≥ college or ≤ high school. Alcohol consumption was indicated as positive for participants who had consumed at least 30 g per day over the last year. The participants’ occupations were categorized as services (which included students, housewives, and the unemployed), agriculture, fishery, or industry. BMI was calculated as weight in kilograms divided by height in meters squared.

### Statistical analysis

All statistical analyses and calculations were performed using SAS V9.2 (SAS Institute). We used the stratification variables and sampling weights designated by the Korean Centers for Disease Control and Prevention.

The baseline characteristics were presented as mean ± standard error (SE), median and range, or frequency and proportions. Comparisons of each variable between the two groups were performed using Student’s *t*-test or modified Rao-Scott chi-square test. Demographic characteristics were analyzed according to eGFR (< 65 or ≥ 65 mL/min/1.73 m^2^), the presence of chronic diseases (diabetes, hypertension, ischemic heart disease, or stroke), and quartiles of blood Mn levels (first lowest quartile vs. other quartiles). The odds ratios (OR) and 95% confidence intervals (95% CI) of the associated factors for reduced eGFR and blood Mn quartiles were estimated using logistic regression. A *p*-value of < 0.05 was considered statistically significant.

## Results

The total number of participants was 3996, and the weighted number was 36,990,120. The mean participant age was 45 ± 0.2 years. The mean blood Mn level in the Korean adult population was 1.34 ± 0.01 μg/dL, and the mean eGFR as calculated by the CKD-EPI equation was 95.1 ml/min/1.73 m^2^. The mean systolic blood, diastolic blood, and arterial pressures were 116.9 ± 0.4 mmHg, 76.7 ± 0.3 mmHg and 88.7 ± 0.2 mmHg, respectively. The baseline clinical characteristics of the participants were shown in Table 1. Blood Mn levels were significantly different in the baseline characteristics of the following variables: sex, smoking exposure, the history of alcohol drinking, and residential area (data not shown). Mn levels were also different according to the presence of chronic disease (Table 2). Participants with diabetes had significantly lower blood Mn levels than those without diabetes (*p* < 0.05). Renal dysfunction and hypertension presented similarly (*p* < 0.05). There was no significant difference in the blood Mn levels of participants with or without ischemic heart disease or stroke.

**Table 1:** **Demographic and clinical characteristics of participants (n = 3996)**^**a**^

**Characteristics**	**% (SE)**
Age, y^b^	45.2 ± 0.2
< 65	86.2 (0.6)
≥ 65	13.8 (0.6)
Sex	
Male	49.5 (0.5)
Female	50.5 (0.5)
BMI, kg/m^2^	
< 25	68.9 (0.9)
≥ 25	31.1 (0.9)
Smoking exposure	
Never	74.3 (0.7)
Former or current	25.7 (0.7)
Alcohol drinking, g/day	
< 30	90.1 (0.6)
≥ 30	9.9 (0.6)
Residence area	
Rural	20.2 (1.8)
Urban	79.8 (1.8)
Education	
≤ High school	70.4 (1.0)
≥ College	29.6 (1.0)
Occupation	
Agriculture & fishery	5.6 (0.6)
Industry	9.2 (0.5)
Services	85.3 (0.8)
GFR, ml/min/1.73 m^2b^	95.1 ± 0.5
Blood pressure^b^	
SBP, mmHg	116.9 ± 0.4
DBP, mmHg	76.7 ± 0.3
MAP, mmHg	88.7 ± 0.2

BMI = body mass index; GFR = glomerular filtration rate; SBP = systolic blood pressure; DBP = diastolic blood pressure; MAP = mean arterial pressure.

^a^Data are given as% (SE) unless otherwise indicated.

^b^Data as the mean ± SE.

**Table 2:** **Characteristics of participants and blood Mn levels according to the presence of chronic diseases**^**a**^

	**Diabetes**			**Renal dysfunction**			**Hypertension**			**IHD**			**Stroke**		
	**Yes (n = 255)**	**No (n = 3735)**	***P *** **value**	**Yes (n = 200**)	**No (n = 3796)**	***P *** **value**	**Yes (n = 1056)**	**No (n = 2921)**	***P *** **value**	**Yes (n = 49)**	**No (n = 3941)**	***P *** **value**	**Yes (n = 43)**	**No (n = 3947)**	***P *** **value**
Age (≥ 65 yrs)	39.1 (3.7)	12.2 (0.6)	< .001	61.6 (4.0)	11.2 (0.5)	< .001	30.8 (1.6)	7.7 (0.6)	< .001	48.7 (8.8)	13.3 (0.6)	< .001	42.8 (9.2)	13.5 (0.6)	< .001
Sex (male)	46.0 (3.4)	49.7 (0.6)	0.31	46.4 (3.6)	49.7 (0.5)	0.38	55.0 (1.5)	47.4 (0.7)	< .001	54.6 (8.0)	49.4 (0.5)	0.52	62.0 (9.4)	49.3 (0.5)	0.19
BMI (≥ 25 kg/m^2^)	49.7 (3.6)	29.9 (0.9)	<.001	47.4 (4.2)	30.2 (0.9)	< .001	46.2 (1.7)	25.8 (0.9)	< .001	49.6 (8.0)	30.9 (0.9)	0.01	35.1 (8.3)	31.1 (0.9)	0.62
Smoking exposure	23.0 (2.8)	25.8 (0.8)	0.34	9.2 (2.2)	26.6 (0.8)	< .001	24.9 (1.5)	26.0 (0.9)	0.54	17.3 (7.7)	25.8 (0.7)	0.92	21.3 (6.5)	25.7 (0.7)	0.53
Alcohol (≥ 30 g/day)	6.3 (1.7)	10.2 (0.6)	0.08	5.7 (1.9)	10.2 (0.6)	0.07	13.5 (1.3)	8.7 (0.6)	< .001	2.1 (2.1)	10.1 (0.6)	0.07	2.8 (2.8)	10.0 (0.6)	0.16
Residence (rural)	20.6 (3.4)	20.2 (1.8)	0.89	20.9 (4.1)	20.1 (1.8)	0.83	22.2 (2.2)	19.5 (1.8)	0.07	29.2 (7.7)	20.1 (1.8)	0.17	24.0 (6.7)	20.2 (1.8)	0.54
Education (≥ college)	16.4 (2.5)	30.4 (1)	< .001	9.3 (2.3)	30.7 (1.0)	< .001	18.7 (1.5)	33.5 (1.1)	< .001	12.1 (5.3)	29.8 (1.0)	0.02	3.7 (2.5)	29.8 (1.0)	< .001
Occupation			0.02			0.93			< .001			< .001			0.95
Services & others	6.1 (1.8)	5.6 (0.6)		5.2 (1.6)	5.6 (0.6)		7.4 (1.1)	5.0 (0.6)		19.0 (7.1)	5.4 (0.6)		5.9 (3.4)	5.6 (0.6)	
Industry	15.2 (3.1)	8.8 (0.5)		8.6 (2.2)	9.2 (0.5)		11.7 (1.1)	8.2 (0.6)		4.2 (2.7)	9.2 (0.5)		7.9 (4.4)	9.2 (0.5)	
Agriculture & fishery	78.8 (3.4)	85.6 (0.8)		86.2 (2.7)	85.2 (0.8)		81.0 (1.5)	86.8 (0.8)		76.8 (7.3)	85.3 (0.8)		86.2 (5.3)	85.2 (0.8)	
Blood Mn (μg/dL)^b^	1.26 ± 0.02	1.35 ± 0.01	0.001	1.28 ± 0.03	1.35 ± 0.01	0.04	1.32 ± 0.01	1.35 ± 0.01	0.04	1.25 ± 0.05	1.34 ± 0.01	0.09	1.35 ± 0.08	1.34 ± 0.01	0.94

IHD = ischemic heart disease; BMI = body mass index; Mn = Manganese.

^a^Data are given as the percentage (SE) of each category in participants with or without chronic disease unless otherwise indicated.

^b^Data as the mean ± SE.

We also evaluated the association of blood Mn level with eGFR and blood pressure. In a linear regression analysis adjusted for age, sex, and BMI, there was no statistically significant association between blood Mn levels and eGFR. However, blood Mn levels were positively associated with systolic blood (β coefficient =1.52, *p* = 0.01), diastolic (β coefficient =1.01, *p* = 0.02), and mean arterial blood pressures (β coefficient =1.26, *p* = 0.004) after adjusting for age, sex, BMI, and presence of diabetes.

We divided blood Mn level into quartiles (Q1–Q4) and analyzed the risk for chronic disease. In a multivariate logistic regression analysis, after adjusting for age, sex, and BMI, participants with blood Mn levels in the higher quartiles had a tendency to have lower odds ratios (OR) for the presence of diabetes with a significant test for trend (*p* = 0.02; Table 3). When the higher quartiles (Q2–4, blood Mn ≥1.060 μg/dL) were compared with the lowest quartile (Q1, blood Mn ≤1.059 μg/dL), the OR for diabetes was 0.652 (95% CI: 0.46–0.92) and 0.589 for renal dysfunction (95% CI: 0.39–0.88, *p* < 0.05) (Table 4). It was well known that diabetes and hypertension were significant risk factors for renal dysfunction; therefore, when adjusted for the presence of diabetes and hypertension, the OR for renal dysfunction was 0.617 (95% CI: 0.41–0.92, *p* < 0.05).

**Table 3:** **Odds ratio and 95**% **confidence interval values of chronic disease presence with blood Mn level after adjusting covariates**

**Mn level quartile (μg/dL)**	**Diabetes**		**Renal dysfunction**		**Hypertension**		**IHD**		**Stroke**	
	**Unadjusted**	**Adjusted**^**a**^	**Unadjusted**	**Adjusted**^**b**^	**Unadjusted**	**Adjusted**^**c**^	**Unadjusted**	**Adjusted**^**b**^	**Unadjusted**	**Adjusted**^**b**^
Q1 (≤ 1.059)	1 (reference)	1 (reference)	1 (reference)	1 (reference)	1 (reference)	1 (reference)	1 (reference)	1 (reference)	1 (reference)	1 (reference)
Q2 (1.060 - 1.277)	0.66 (0.45, 0.97)	0.65 (0.43, 0.99)	0.52 (0.34, 0.81)	0.51 (0.31, 0.84)	1.09 (0.86, 1.37)	1.21 (0.93, 1.57)	1.03 (0.41, 2.57)	1.04 (0.38, 2.80)	0.92 (0.37, 2.30)	0.91 (0.35, 2.39)
Q3 (1.278 - 1.559)	0.86 (0.57, 1.30)	0.75 (0.49, 1.15)	0.78 (0.50, 1.20)	0.64 (0.39, 1.07)	1.15 (0.92, 1.44)	1.16 (0.90, 1.50)	1.49 (0.62, 3.59)	1.41 (0.55, 3.62)	1.22 (0.50, 3.00)	1.21 (0.48, 3.07)
Q4 (≥ 1.560)	0.53 (0.35, 0.80)	0.53 (0.34, 0.84)	0.67 (0.43, 1.05)	0.72 (0.43, 1.20)	0.86 (0.69, 1.07)	1.07 (0.83, 1.39)	0.47 (0.17, 1.36)	0.55 (0.18, 1.65)	0.94 (0.30, 2.94)	1.22 (0.39, 3.80)
*P* value	0.01	0.04	0.03	0.06	0.07	0.49	0.11	0.26	0.93	0.92
*P* for trend	0.02	0.02	0.22	0.28	0.28	0.65	0.41	0.60	0.94	0.62

IHD = ischemic heart disease; Mn = manganese.

^a^adjusted for age, sex, body mass index and hypertension.

^b^adjusted for age, sex, body mass index, diabetes and hypertension.

^c^adjusted for age, sex, body mass index and diabetes.

**Table 4:** **Odds ratio, 95**% **confidence interval and prevalence of chronic diseases comparing the higher quartiles with the lowest quartile of blood Mn level after adjusting covariates (Q1 vs Q2–4)**^**a**^

**Parameter**	**Odds ratio (95% confidence interval)**		**Prevalence (%, SE)**		
	**Unadjusted**	**Adjusted**	**Q1**	**Q2-4**	***P *** **value**
Diabetes	0.68 (0.50, 0.94)	0.65 (0.46, 0.92)^b^	7.6 (0.9)	5.3 (0.5)	0.02^b^
Renal dysfunction	0.65 (0.46, 0.92)	0.62 (0.41, 0.92)^c^	6.8 (1.0)	4.6 (0.5)	0.02^c^
Hypertension	1.03 (0.85, 1.24)	1.15 (0.93, 1.42)^d^	25.6 (1.6)	26.2 (1.0)	0.77^d^
IHD	1.00 (0.46, 2.16)	1.04 (0.45, 2.40)^c^	1.2 (0.4)	1.2 (0.2)	0.99^c^
Stroke	1.03 (0.48, 2.20)	1.11 (0.51, 2.42)^c^	0.8 (0.2)	0.8 (0.2)	0.94^c^

IHD = ischemic heart disease.

^a^Blood manganese quartiles (μg/dL): Q1 ≤ 1.059, Q2 1.060 - 1.277, Q3 1.278 - 1.559, Q4 ≥ 1.560.

^b^adjusted for age, sex, body mass index and hypertension.

^c^adjusted for age, sex, body mass index, diabetes and hypertension.

^d^adjusted for age, sex, body mass index and diabetes.

The prevalence of diabetes was significantly higher in the lowest quartile (Q1) when compared with the higher quartiles Q2–4 (7.6% *vs.* 5.3%, *p* = 0.02). Similarly, the prevalence of renal dysfunction showed a significantly higher value in the lowest quartile (Q1) than in the higher quartiles (6.8% *vs.* 4.6%, *p* = 0.02; Table 4).

## Discussion

This study was the first to examine the effects of blood Mn on chronic diseases in the general population and determine that blood Mn levels affected the morbidity of chronic diseases. It was found that low blood Mn levels increased the risk and prevalence of diabetes and renal dysfunction, suggesting that blood Mn deficiency might be involved in the pathophysiological processes of diabetes and renal dysfunction.

Mn is stored primarily in skeletal bones and tissues rich in mitochondria. However, no existing biomarker can reliably determine the exact level of Mn accumulated in the body. Because there is a discrepancy in the half-life of Mn in the tissues and blood, Mn levels in red blood cells or whole blood are believed to be more reliable than plasma Mn for measuring Mn accumulation in the body [3]. For practical reasons, whole blood samples have been used as an exposure biomarker of Mn inhalation in most epidemiological studies [3] and were used accordingly in the present study to measure blood Mn.

The importance of Mn in animals was first reported in 1931, but there has been little evidence of its deficiency in humans [1]. In animal models, some of the abnormalities seen in Mn deficiency relate to skeletal abnormalities, changes in circulating HDL cholesterol, and reproductive failure [4]. Some studies have proposed that Mn may influence high glucose conditions. A relationship between Mn and pancreatic function was first seen in 1962 when the blood glucose level dropped in a diabetic patient orally administered Mn. It was postulated that Mn might accelerate cellular glucose uptake by potentiating insulin action and that Mn might act on the pancreas by stimulating the release of stored insulin into the bloodstream or by inhibiting the release of glucagon [10]. In addition, Baly *et al.* demonstrated that Mn deficiency resulted in decreased synthesis of pancreatic insulin, enhanced insulin degradation, and lowered insulin secretion in an Mn-deficient animal model [11]. It was also suggested that Mn deficiency could affect glucose transport and metabolism in adipose cells [12]. A recent experiment demonstrated *in vitro* and *in vivo* that Mn^2+^ supplementation lowered the risk of endothelial dysfunction in diabetes [13].

In the present study, we found a statistically significant relationship between blood Mn levels and diabetes in a representative Korean population. Blood Mn levels were lower in participants with diabetes but not in participants with ischemic heart disease or stroke. Furthermore, the prevalence of diabetes significantly increased in participants with blood Mn levels in the lowest quartile. The pathophysiological mechanism of these results remained unclear. However, the present findings were consistent with previous reports of hypoglycemia induced by low-dose Mn intake in diabetic patients, as well as changes in insulin metabolism in Mn-deficient animal models [10,11,14]. Further studies into the association of blood Mn and insulin metabolism or glucose control status (such as hemoglobin A1c) are needed.

Prior to this study, no clear relationship between Mn and diabetes had been found, and the few epidemiologic studies that addressed this issue in humans were controversial. With limited comparable data, one study into the general elderly population of the Czech Republic found no association between blood Mn and diabetes; instead, seniors with atherosclerosis had higher Mn levels than those without [15]. Three other studies linked low Mn levels with diabetes. Kazi *et al.* showed that diabetic patients of both genders in Pakistan had significantly lower blood and hair levels of Mn than those in the controls [16]. Ekmekcioglu *et al.* found decreased Mn levels in the whole blood and erythrocytes of diabetic individuals [17]. The most recent study revealed Mn deficiency in both type 1 and type 2 diabetic patients with respect to control subjects [18].

Many studies have focused on environmental Mn toxicity rather than on its deficiency and metabolism. Mn toxicity in humans primarily occurs as a consequence of chronic inhalation of high concentrations of airborne Mn-containing particles, linking symptoms of Mn toxicity mainly to miners as well as ferroalloy and battery manufacturing workers [5]. In Korea, Mn may be exposed occupationally or environmentally. Typical toxicity symptoms resemble those of idiopathic Parkinson’s disease and include tremor, rigidity, bradykinesia, and posture instability. Patients may also display neuropsychological difficulties that include apathy and even psychosis as Mn affects the dopaminergic systems with a neuropathology that closely resembles Parkinson’s disease [19]. Most studies have examined outcomes following relatively high-level acute or chronic exposure to Mn. Less is known about the effects of chronic exposure to lower levels of Mn or the threshold levels sufficient for altering cognitive and motor function [19].

Oxidative stress and inflammation play a major role in the progression of renal damage in chronic kidney disease (CKD). Mn is a potent antioxidant and cofactor of the enzyme MnSOD, which is the main antioxidant enzyme in the mitochondria responsible for protecting the cell from reactive oxygen species (ROS) by scavenging mitochondrial superoxides. Various studies using Mn^2+^ have linked its effects with the function and role of MnSOD. The results in this study showed a significant association of blood Mn level with the prevalence and risk of renal dysfunction after adjusting for diabetes and hypertension in logistic regression analysis, although not in linear regression. Because the KNAHNES was conducted in the general population of Koreans, we projected that including too small a size of subjects with moderate to advanced renal dysfunction may influence the statistical result. Thus, we cautiously postulated that subgroups with lower blood Mn levels may have lower MnSOD activities, possibly making them more sensitive to oxidative stress. Further studies are needed to verify this hypothesis.

High blood Mn levels were found, in this study, to be consistently associated with high systolic, diastolic, and mean arterial blood pressures after adjustment, although no differences in the prevalence of hypertension were found between the lower and higher blood Mn quartile subgroups. These results may have been conflicting as hypertension was partially dependent on self-reported data. Nevertheless, these findings were somewhat compatible with a previous study based on the KNHANES 2008, which reported that blood Mn levels may have been associated with an increased risk of hypertension [20]. The study assumed that chronic Mn accumulation in the mitochondria affected MnSOD activity, resulting in an abnormal response to the mechanism that blocked oxidative stress in the mitochondria and protected against endothelial dysfunction. The exact pathophysiology of Mn on blood pressure is unclear and remains to be elucidated.

This study had some limitations. First, it was cross-sectional, making it difficult to establish a cause-and-effect relationship. Diabetes appeared to have caused lower Mn based on data showing low stores of albumin and other binding proteins in diabetic patients [21], suggesting that Mn and diabetes may affect each other. Further analysis with a longer follow-up period is needed to support the clinical importance of blood Mn levels in diabetes. Second, our regression models were not fully adjusted for potential confounders, such as serum iron or albumin levels, which are the main Mn-binding proteins in the blood. Furthermore, we did not adjust for other metals with the same valence states as Mn, including copper and calcium, which may affect blood Mn levels and other chronic diseases. There may have been participants with chronic diseases without Mn readings, which may explain the lack of a significant association between blood Mn levels and ischemic heart disease or stroke. Last, insufficient information was available to specify whether the diabetes subgroups were type I or II. Despite these limitations, the study presented solid findings as it was performed on a representative sample of the general Korean population with strict quality control of the study procedures of the KNHANES.

## Conclusion

The prevalence of diabetes and renal dysfunction decreased in participants with high blood Mn levels, suggesting that blood Mn may have some role in glucose homeostasis and renal function. Further studies are needed to determine any protective effects of Mn on diabetes and renal dysfunction.

## Competing interests

The authors declare that they have no competing interests.

## Authors’ contributions

SJS had full access to all the data in the study and took responsibility for the integrity of the data and accuracy of the data analysis. Study concept and design: SJS, SC, and JHC. Acquisition of data: SJS, ESK, HEY, and SJK. Statistical analysis and interpretation of data: SJS, SC, and JHC. Draft of the manuscript: SJS and ESK. Critical revision of the manuscript for important intellectual content: CWP and YSC. All authors read and approved the final manuscript.

## Pre-publication history

The pre-publication history for this paper can be accessed here:

http://www.biomedcentral.com/1472-6823/14/24/prepub
